# Nursing Minimum Datasets in Long-Term Care Settings: Scoping Review

**DOI:** 10.2196/68670

**Published:** 2025-10-14

**Authors:** Sarah Milkov, Lara Linders, Alina Napetschnig, Wolfgang Deiters, Erika Sirsch, Daniela Holle

**Affiliations:** 1 Department of Nursing, Midwifery, and Therapy Sciences, Bochum University of Applied Sciences Health Campus, Gesundheitscampus 6-8, Bochum, 44801, Germany, 49 23477727701; 2 Department of Health Sciences, Bochum University of Applied Sciences Health Campus, Bochum, Germany; 3 Institute of Medical Education, University of Duisburg-Essen, Essen, Germany

**Keywords:** nursing minimum dataset, long-term care, care home, databases, nursing informatics, nursing, standards

## Abstract

**Background:**

Standardized and structured data collection is necessary in the health care sector to advance nursing research, enable the comparison of practice-based data, and optimize the potential of technological innovations and digitalization. This can be supported by nursing minimum datasets (NMDSs).

**Objective:**

This scoping review aims to present the state of research on NMDSs in long-term care settings.

**Methods:**

Articles addressing NMDSs in long-term care published in the PubMed, CINAHL, Embase, or Digital Bibliography & Library Project databases up to September 2024 were included. Additionally, forward and backward citation tracking and manual searches of original records were conducted. All types of articles were included, with no time limit, and the articles had to be in English or German. The selected sources were screened and evaluated in a double-review process. Evaluation was carried out using a qualitative content analysis approach, supplemented by inductive and model-based categorization, and concluded with a final narrative synthesis.

**Results:**

A total of 36 sources covering 9 NMDS projects or initiatives were included. Most of the included articles (15/36, 42%) were published between 2004 and 2015. The United States accounted for the largest share (16/36, 44%) of the country of origin. The topic of NMDSs has gained more relevance in recent years, with 5 sources from 2022 to 2024 being found. Most publications were overview articles (15/36, 42%), followed by reviews and discussion papers (5/36, 14% each), highlighting the literature’s predominantly conceptual and discursive focus. Various types of NMDSs were identified, including country-specific, topic-specific, and international adaptations of the US NMDS systems. The content of the NMDSs could be categorized as patient, interpersonal, or institutional data. The most comprehensive information is available on the US NMDS. Many initiatives were described but few have been developed or are currently in use. The literature included recommendations at the clinical, scientific, and administrative levels, emphasizing standardization, stakeholder involvement, and using NMDS data to improve care practices and policies.

**Conclusions:**

NMDS initiatives are becoming increasingly important in the context of digitalization, demographic change, and legislative developments, especially in Europe. Existing NMDSs primarily focus on patient data, and nursing interventions, outcomes, and the perspectives of individuals in need of care have so far received little attention. A lack of standardized descriptions and scientifically usable content hinders comparability and further development, underscoring the need for legal frameworks and stronger involvement from health care practitioners and researchers.

## Introduction

### Background

In this scoping review, we focus on the concept of nursing minimum datasets (NMDSs), which were defined by Werley et al [1] as “a minimum set of items of information with uniform definitions and categories concerning the specific dimension of nursing which meets the information needs of multiple data users in the health care system.” They can act both as tools and strategies to establish and standardize essential data elements for widespread use among nurses in diverse health care settings. Their broad applicability also makes them suitable for use by other health care professionals, researchers, and systems [2]. The development and introduction of NMDSs involves several objectives, including the comparison of nursing data across clinical populations, settings, geographic areas, and time; the description of the nursing care of clients and their families; the demonstration of trends regarding nursing care; the promotion of nursing research; and the provision of data about nursing care to influence and facilitate health policy decision-making [1]. An NMDS can be understood as a nursing terminology that also supports the standardization of nursing documentation and thus the use of digital health care systems [3].

Given its wide relevance across different contexts, this review focuses on NMDSs in long-term care settings. Owing to demographic changes in many European countries, there has been an increase in the number of older individuals with complex care needs, which the COVID-19 pandemic only further exacerbated, leading to more patients in long-term care facilities, and this trend is expected to increase in the future [4]. Here, we refer specifically to residential homes, nursing homes, and rehabilitation facilities for older adults.

### Need for NMDSs in Long-Term Care

The long-term care sector requires structured and standardized data to provide consistent, comparable, and evidence-based care, which an NMDS can effectively address [1]. This type of data is essential for nursing practice. It enables management to plan staffing and allocate resources based on reliable information, thereby improving the quality of care [5]. Structured data are also indispensable for nursing research, as Werley et al [1] emphasized in their definition of the NMDS. Additionally, the NMDS plays a key role in an increasingly digitalized care infrastructure. Although digitalization is increasingly advanced through the expanded use of electronic health records, the COVID-19 pandemic has also highlighted significant and persistent gaps in long-term care [6,7]. To address these issues, legislative measures regarding digitalization in the health care sector have been enacted, and measures in Germany serve as an example. Legislative measures such as the Digitale-Versorgung-und-Pflege-Modernisierungs-Gesetz Digitalgesetz, and Gesundheitsdatennutzungsgesetz aim to promote the use of digital applications to improve patient care, integrate electronic health records, and facilitate access to health data for research purposes [8-10].

Improvements to the structure and standardization of data will facilitate better use of data for research and support legislative measures aimed at improving health care delivery. To achieve these benefits, however, NMDS data must be valid and comparable; missing or inconsistently documented information impairs the reliability of the results and the comparability of the data. Therefore, the availability and retrievability of NMDS elements in patient records is essential [11].

### Prior Work and Initiatives

The literature addresses the history, development, content, and evaluation of NMDSs from the 1980s to the present day [1,12,13]. Recently, a mapping review (preprint) was published that is specific to published literature focusing on original research that uses data from minimum datasets (MDSs) in care homes [14]. Additionally, an umbrella review for NMDSs in the hospital setting by Freguia et al [2] aims to summarize the evidence available on NMDSs. However, to our knowledge, no review has provided a comprehensive overview of existing NMDSs and initiatives for developing them over time in the context of long-term care. Before starting our research, we searched portals such as PROSPERO for reviews on this topic but found none.

Different initiatives, for example, the German Medical Informatics Initiative (MII) and the European Health Data Space, aim to integrate health data from different institutions to facilitate the exchange of data and enable the analysis of data for various purposes (eg, artificial intelligence–based research for pattern recognition) [15,16]. The MII, for example, has developed an MII core dataset that serves as a standardized dataset (technical database model) for integrating medical data [15]. The federally funded research project Entwicklung eines Pflege-Kerndatensatzes und Aufbau eines intersektoralen Pflegedaten-Repository (PFLIP; development of a nursing minimum data set and establishment of an intersectoral nursing data repository in English) aims to enhance fall detection by integrating a nursing-relevant MDS, which is defined and supplemented alongside medical data from the MII core dataset [17].

Owing to the broad time range of published sources and the variety of information, it is difficult to obtain a comprehensive overview of the literature. This scoping review aims to provide a broad overview of international NMDSs and their contents and recommendations and to place them in the current context.

This review is a scoping review [18]. Compared with other review types, a scoping review is characterized by its ability to map the key concepts of a research area or to define the content boundaries of a topic. Scoping reviews also provide an overview of the available evidence, regardless of its quality, which is suitable for the broad topic of NMDSs [18].

### Objectives

The aim of this review is to present the current state of research on NMDSs in long-term care settings. Specifically, it addresses the following research questions:

1. What NMDSs or initiatives for the development of NMDSs in long-term care exist? 
2. What nursing data do these NMDSs contain? 
3. What recommendations are made in the literature regarding NMDSs in long-term care settings?

## Methods

To ensure the high quality of the review, the Joanna Briggs Institute Manual for Scoping Reviews was used as a guideline [18]. In addition, the PRISMA-ScR (Preferred Reporting Items for Systematic Reviews and Meta-Analyses Extension for Scoping Reviews) guidelines (Checklist 1) were followed [19].

### Eligibility Criteria

Articles had to be available in English or German. No time limit regarding the publication date of the articles was set, as the topic is relevant both in the past and today. In addition, all article types were included, as the review is intended to provide an overview of the state of research from all publication types.

The inclusion and exclusion criteria were defined in advance, and articles were selected on the basis of these criteria after the abstracts were read. The criteria are listed in Textbox 1 according to the objective and research questions.

Textbox 1. **Inclusion and exclusion criteria.**

**Inclusion criteria**

- Population: adults in need of care and care home residents
- Concept: nursing minimum datasets
- Context: long-term care setting

**Exclusion criteria**

- Population: children and adolescents
- Concept: minimum datasets without reference to nursing
- Context: acute or outpatient care setting

### Population

The content of the identified articles had to be related to adults aged ≥18 years and in need of care. We excluded articles that included children or adolescents as a target group.

### Concept

Articles that addressed the concept of NMDSs were included. The articles had to address this topic primarily and in accordance with the defined research questions. Articles that addressed the concept of NMDSs incidentally, for example, the use of an NMDS as a survey instrument, or articles that referred to MDS without a specific nursing reference were excluded.

### Context

The NMDS set out in the articles had to be related to the setting of long-term inpatient care. This setting includes, for example, nursing homes, retirement homes, or rehabilitation facilities. We excluded articles related to other care settings, such as hospitals or outpatient care.

### Information Sources

The well-established medical and nursing scientific databases PubMed, CINAHL, and Embase were searched to identify important sources. To achieve the largest possible number of hits, a database in the field of informatics named the Digital Bibliography & Library Project database was included. The search began in February 2023 and was updated in September 2024. The search results of each database were added to a reference management system (EndNote 21, Clarivate). The search strategy was developed jointly by the review team and was prepared in advance with a librarian for quality assurance purposes.

### Search

The review team carried out a systematic literature search. An overview of the evidence selection steps and the results are shown in Textbox 2. The team used a combination of the following search terms, with the Boolean operators AND between concepts and context and OR between synonyms.

Textbox 2. **Keywords for the database search.**

**Concept**

- *Core data set* OR
- *Minimum data set* OR
- *Minimum dataset* OR
- *Nursing minimum data set* OR
- *Nursing minimum dataset* OR
- *MDS* OR
- *NMDS* OR
- *NHMDS* OR

**Context**

- *Longterm care* OR
- *Long-term care* OR
- *Care home* OR
- *Nursing home* OR
- *Carehome* OR
- *Retirement home* OR
- *Homes for the elderly* OR
- *Care facility* OR
- *Nursing facility* OR
- *Psychiatric facility* OR
- *Rehabilitation care* OR

First, a PubMed database search was carried out. The corresponding search query was subsequently adapted for the other databases [20]. In addition to whole words, common abbreviations (acronyms) were included. The exact application and adaptations to the respective databases can be found in Multimedia Appendix 1. Following the systematic literature search, forward and backward citation tracking was carried out via Google Scholar [21]. For articles that were not available to the research team, access was requested from the authors. Additionally, manual web searches for original data records of the NMDSs were conducted.

### Selection of Sources of Evidence

First, duplicates were removed. This was done using the Find Duplicates function in the EndNote program. The duplicates were displayed, allowing the reviewer to select and remove them. Some duplicates were also identified during the screening process and removed manually using the reference management program. Then, the abstracts of all potentially relevant articles were screened in a double-review process. Sources were evaluated independently by pairs of reviewers according to predefined inclusion and exclusion criteria. Five reviewers were involved in the screening and evaluation process. First, a reduced number of articles were read. Then, all reviewers discussed and optimized the inclusion and exclusion criteria. The results were recorded and made available to each reviewer. In the second round, pairs of reviewers were formed to further analyze the abstracts, ensuring that each abstract was read by 2 reviewers to achieve the highest possible level of agreement. If there was disagreement about the inclusion or exclusion of an article, the reasons were discussed. If no agreement was reached, a third reviewer was consulted. The full texts identified were subsequently screened using the same procedure.

### Data-Charting Process

The identified articles were entered into the MAXQDA (VERBI Software) literature processing program, where the content was coded and analyzed. The research team developed a data-charting form with corresponding categories and codes and initially tested it on 10% of the articles with 1 reviewer. The form included predefined categories derived from the research question and open areas for developing categories related to the research question. The results were then discussed, and the categories and codes were adapted by the entire review team. One reviewer carried out the coding and analysis, and a second reviewer checked it. A complete list of all categories can be found in Multimedia Appendix 2.

### Data Items

First, the characteristics of the included articles were collected using a predefined data-charting form. These characteristics included author, country of origin, year, journal, article type, and objectives. In contrast, categories addressing the first research question were formed inductively according to the qualitative content analysis by Mayring [22]. NMDSs were specifically identified from the sources. The respective context of origin was then systematically analyzed. As part of the inductive coding process, the NMDS concepts were classified into categories, which are presented in the Results section.

For the second research question, the contents of the NMDSs were categorized according to a structural model for NMDS data, as described by Werley et al [1], including the following top categories: patient data, interpersonal data, and institutional data (Multimedia Appendix 2). To this end, aspects were extracted from the literature and classified according to the model. 

For the third research question, the categories from the study by Freguia et al [2] were used as a guide for the different recommendation categories, which included overall, clinical, research, and managerial recommendations. The various recommendations from the articles were assigned to the appropriate category according to their context of application.

This was followed by a narrative synthesis, which brought together the results from the articles with a focus on summarizing all relevant information in 1 category. This synthesis provided an overall overview of the identified NMDSs, their contents, and the recommendations given. This approach enabled the presentation of the most important findings in a clear and concise manner and illustrated the state of research in the respective areas.

### Critical Appraisal of Individual Sources of Evidence

As this article met the requirements of a scoping review, no critical appraisal of the included studies was conducted.

## Results

### Selection of Sources of Evidence

**Figure 1.**
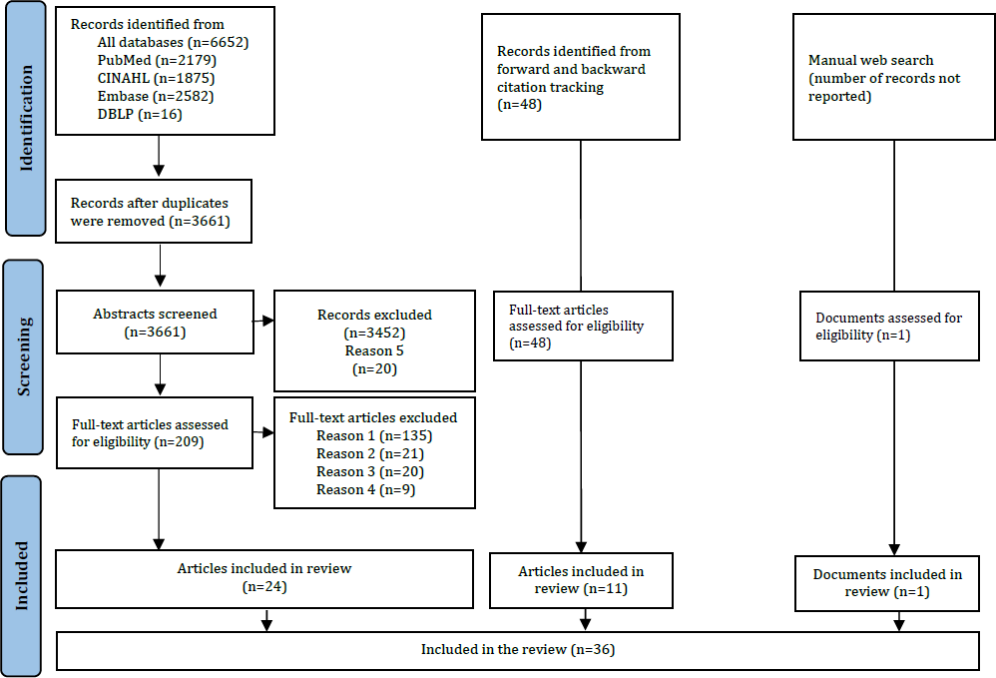
PRISMA flowchart of the included studies. Reasons for exclusion: (1) thematically inappropriate; (2) ambulatory setting; (3) hospital setting; (4) different languages; (5) no full text available. DBLP: Digital Bibliography & Library Project.

In the systematic literature search, a total of 3661 abstracts and 209 full texts were screened by the review team. Articles were excluded for the following reasons: thematic deviation from the topic, focus on a setting other than inpatient long-term care, unavailability of full text, or publication in a language other than English or German. Through forward and backward citation tracking, 48 articles were identified as relevant, of which 11 were included. Some articles were not available for analysis despite requests to the authors. As a result of the study selection process, 36 sources from the systematic literature search were included in the review, of which 24 (67%) resulted from the database research, 11 (31%) from the forward-backward citation tracking, and 1 (3%) from the manual internet research. An overview of the process can be found in the PRISMA (Preferred Reporting Items for Systematic Reviews and Meta-Analyses) flowchart (Figure 1).

### Characteristics of the Sources of Evidence

Multimedia Appendix 3 [1,5,7,12,14,23-53] presents the characteristics of the sources of evidence, including the article type, publishing journal, and main objectives. Of the 36 included sources, most articles were published in 2004 (n=4, 11%); 18 (50%) were published in 2003, 2007, 2012, 2015, and 2024 (n=3, 8% articles each); 2 (6%) each in 2009, 2013, and 2023; and 1 (3%) each in 1991, 1992, 1995, 1998, 1999, 2000, 2010, 2011, 2017, 2018, and 2022.

In terms of countries of origin, the United States has the largest share with 16 (44%) out of 36 articles. Canada, Germany, the United Kingdom, and transnational articles follow with 3 (8%) out of 36 articles each. Switzerland is represented with 2 (6%) out of 36 articles. The remaining countries, including Taiwan and the Netherlands, as well as cross-country articles and additional contributions from the United States and the United Kingdom, have 1 (3%) out of 36 articles each.

The article types are predominantly classified as overview articles, with 15 (42%) out of 36 articles. This is followed by review and discussion articles, with 5 (14%) out of 36 articles each. The least represented article types are book sections, editorials, comments, letters to the editor, personal views, clinical reviews, mixed methods studies, mapping reviews, development studies, and research articles, each comprising 1 (3%) out of 36 articles.

### Results of Individual Sources of Evidence

The various sources of evidence were analyzed on the basis of the research questions. There were 9 NMDSs identified in the 36 sources. The results are presented below.

#### NMDS Projects or Initiatives

In relation to the first research question, which concerns existing NMDS projects or initiatives in long-term care, the results are shown in Multimedia Appendix 4. We identified different types of NMDSs: country-specific NMDSs or projects, topic-related NMDSs for research and adaptations, or translations of existing NMDSs. The first NMDS, which was developed and implemented in the United States, dates back to 1990 and is up to date [1,23,24]. As shown in Multimedia Appendix 4, there were many initiatives related to translating the US NMDS into other languages and adapting the American concept to other countries [25-33]. The most current evidence for such transformations originates from Switzerland and Taiwan [29,34]. In addition to the US NMDS, there are results from different countries. In their article from 1998, Goossen et al [35] described an initiative to build an NMDS in the Netherlands, first used in the hospital setting and then later expanded to other nursing settings. We found no further work on the development of NMDSs for the long-term care setting in the Netherlands. In their review, Goossen et al [35] mentioned 2 projects for the development of NMDSs in all settings: the Telenurse project (Europe) and the Health Information: Nursing Components (HI:NC) project (Canada) [35]. No further literature was found for these projects, and after corresponding with the authors of the review, we assume that there have been no recent developments on these projects. For Canada, there is also an NMDS for the inpatient rehabilitation care setting. Since 2003, the use of the National Rehabilitation Reporting System (NRS) has been mandated in all Ontario inpatient adult rehabilitation facilities [37]. In the United Kingdom, there is an ongoing study for developing an NMDS for the long-term inpatient setting called the *Developing Resources and Minimum Data Set for Care Homes’ Adoption* (DACHA) study [7,12,14,38,39]. A sizable amount of recently published literature was found regarding the UK MDS, which aims to improve the accessibility and interoperability of routinely collected data in care homes and standardize the way in which data on residents’ needs and events are collected [39]. Furthermore, there are NMDSs for research related to the following topics: the MDS for nutritional intervention studies, the MDS for research studies on falls and osteoporosis, and the MDS for intervention studies on type 2 diabetes. These datasets are related to special areas of nursing research with clear implications for inpatient long-term care practices. All of this evidence dates from 2004 to 2007, and no further research was found about the datasets [40-42].

#### Contents of the NMDSs

Regarding the second research question, when illustrating the contents of the NMDSs, the original data records were used where available. This affected only the US NMDS, and the contents of the others were derived from the literature. Twenty-four articles, as well as 1 additional document (MDS 3.0 item set by the Centers for Medicare & Medicaid Services), included contents of NMDSs [43]. Multimedia Appendix 5 shows the results regarding the contents of the identified NMDSs. To simplify the presentation of the extensive NMDS content, we followed the classification used by Werley et al [1]: (1) patient data, (2) interpersonal data, and (3) institutional data. No information on the content of the NMDS from the Netherlands was found in the literature [35].

##### Patient Data

The patient data category contains data on demographics, physiological and psychosocial factors, diagnoses, and patients’ perceptions and goals (Multimedia Appendix 5). The majority of the elements were identified from the US NMDS, particularly in the sections on physiological and psychosocial factors and diagnoses. The physiological and psychosocial factors and diagnoses mainly included information on hearing, speech, and vision; cognitive patterns; mood; behavior; bladder and bowel; swallowing and nutritional status; oral and dental status; and skin conditions (Multimedia Appendix 5) [1,23,26,27,29,43-52]. A series of assessments is described in the MDS 3.0 item set, for example, to measure and document the characteristics of certain states and conditions [43]. The NRS (Canada) includes 2 patient data items: patient demographics and functional ability; however, it is the only dataset that is related to the rehabilitation setting [37]. The topic-related NMDSs for research contained demographic data as well as information on physical and psychosocial factors and diagnoses according to the thematic focus of the datasets [40-42].

##### Interpersonal Data

The interpersonal data category contains all identified elements related to care actions or orders, including nursing interventions, nursing and medical orders, and nursing or client outcomes. Notably, few nursing interventions and outcomes were mentioned in the US NMDS. The NMDS for fall and osteoporosis intervention studies included various outcomes, as the dataset should define outcome measures for studies [41]. For the Telenurse and HI:NC projects, the general categories identified included diagnoses, nursing interventions, and nursing outcomes [35].

##### Institutional Data

The institutional data category contains all formal aspects of the respective institutions, such as size; location or staffing data; and episodic information, for example, admission and discharge of residents. These aspects can be found in the US NMDS, Telenurse project, HI:NC project, UK NMDS, and NMDS for nutritional intervention studies (Multimedia Appendix 5).

### Recommendations Related to NMDSs

Multimedia Appendix 6 relates to the third research question, which addresses the recommendations made in the articles on NMDSs. The categories *overall*, *clinical*, *research*, and *managerial* were selected on the basis of the descriptions by Freguia et al [2]. Recommendations related to the detailed thematic content of the NMDSs were not included. Most recommendations were at the *overall* and *clinical* level, with the fewest at the *research*” level. There were no recommendations for the Telenurse and HI:NC projects or for topic-related NMDS projects [35].

### Overall Recommendations

Articles on NMDSs from both the United States and the Netherlands make general recommendations for the respective MDSs. One issue was the uniformity of documentation to ensure consistent and standardized nursing data. This is described by, for example, Dougherty et al [45], who used the example of improving the accuracy of diagnoses and noted that uniform coding is crucial to accomplishing this. In addition, Goossen et al [35] highlighted the importance of the accurate definition of items and variables. Furthermore, researchers recommended that data be available to support policy decisions [1,35]. With respect to the general content of NMDSs, a study by Hanratty et al [14], from the United Kingdom, emphasized that the content of NMDSs should focus on residents’ needs. NMDSs should also reduce the amount of documentation required in nursing. Additionally, stakeholders, including care home residents, should be involved in the evidence-based development of NMDSs, and NMDS data should serve as a basis for nursing research. Finally, the issues of privacy and security in data exchange should also be considered [7].

### Clinical Recommendations

The clinical recommendations focused on the involvement of clinicians or physicians regarding results for residents obtained from NMDSs [46,48,50]. This involvement is particularly important at points where the patient’s voice is involved so that the corresponding data can be used correctly [48]. In addition, training for staff was recommended at several points, including the integration of the patient voice and the coding of standardized data [1,23,45,52]. This recommendation was supported by Goossen et al [35], who recommended a multidisciplinary approach, and by Wells et al [37], who advocated for including the physician’s perspectives on actual and potential educational and policy uses of NRS data.

### Research Recommendations

At the research level, Wells et al [37] stated that nursing research should focus more on the older adult population. Goossen et al [35] provided recommendations on how research can ensure good international comparability [37]. One focus of the study by Killett et al [12] was the importance of bridging the divide between research and practice in social care by integrating practical knowledge and fostering closer collaboration between researchers and practitioners, thereby enhancing knowledge transfer and minimizing unintended consequences in implementation.

### Managerial Recommendations

At the managerial level, it was recommended that attending physicians and medical directors collaborate with nursing home leaders to establish mechanisms for effectively communicating the results of specific patient data to ensure comprehensive care. Furthermore, nurse administrators should advocate for the abstraction of core nursing data across various care settings, which will not only facilitate improved public health nursing practices but also enhance research opportunities by providing comparable data for computerized nursing information systems [1,48].

## Discussion

### Principal Results

Regarding the first research question, the results align with current developments in the health care sector. The far-reaching consequences of the COVID-19 pandemic, demographic changes in Europe, and increasing digitalization in the health care sector could help explain why NMDSs have recently gained attention [4]. This is also supported by recent studies, for example by Tschorn et al [54] and Netzband et al [55], which demonstrate how standardized nursing datasets can be developed to serve as a basis for nursing decision support systems and the use of artificial intelligence. Thus, an NMDS serves as a key strategic element for digital transformation and data-based decision-making in nursing care.

As mentioned in the Background section, Germany is legislating the topic of digitalization in the health care sector, and new federally funded projects and initiatives on this topic have been introduced [5-7]. Notably, the United States also has legal foundations for this concept. As the topic of NMDSs is currently gaining importance in Germany and NMDSs have been established in the United States for decades, it can be assumed that regulatory measures may help to ensure the consistent implementation of NMDSs. In view of inadequacy, the process of developing past NMDSs must also be considered. It can be assumed that practitioners were not sufficiently involved in developing previous datasets. However, to develop an NMDS, it is essential to involve all relevant stakeholders, including care professionals and management, to ensure practicability and acceptance [7]. As management is responsible for implementing an NMDS, they must be interested in and committed to using the NMDS as a tool for mapping care needs, needs-based staff planning, and transparency regarding the quality of care in long-term care.

The answer to the second research question suggests that previous NMDSs had a greater focus on patient data, with the inclusion of residents’ perspectives varying across different datasets. In particular, the UK NMDS focuses on “residents’ needs,” reflecting the growing importance of incorporating patient and resident perspectives in health care research in the United Kingdom. This approach is also recommended for NMDS development [14,56]. It also found its way into the US NMDS, demonstrating that newer articles on the US NMDS also explicitly address the ”resident voice” [23]. Data on the impressions, thoughts, emotions, and relationships of residents are difficult to standardize, which could explain why these were not accounted for to a sufficient extent in the development of the NMDSs from the outset. Due to the large amount of medical patient data and lesser focus on the nursing perspective in some NMDSs, nursing research may be limited. For example, intervention or outcome studies may not be feasible. In this sense, the general quality of the NMDS content should also be considered. For research purposes, the content should be scientifically evaluable, for example, using internationally recognized care diagnoses. We also want to reiterate the importance of valid and comparable data to achieve relevant results [11].

In terms of the third research question, the topic of research was given less consideration in the recommendations than in the other categories. This finding implies a need for further research regarding the extent to which NMDSs have contributed to nursing research from the outset. The recommended interdisciplinarity and collaboration between medicine and nursing indicate that these sectors should work in a complementary manner. To obtain a better understanding of the health and nursing situation, especially for older people, it would be beneficial to integrate both medical and nursing data (these data are being pursued in Germany through the MII core dataset and the research project PFLIP) [15,17]. An NMDS could form the basis of a nursing-specific dataset a comprehensive view of medical and nursing elements. Furthermore, cooperation between research and practice is essential, which underscores the importance of incorporating a practical perspective into NMDSs [7]. In addition, establishing a structural framework analogous to legally anchored, standardized medical data systems for service documentation and billing (eg, diagnosis-related groups) would be useful. The example from the United States illustrates this point.

### Limitations

This scoping review has several limitations. First, owing to the methodology of this type of article, all types of sources were included, regardless of their scientific quality. Second, not all full texts that would have been included after screening the abstracts were available. Thus, some important information may not have been included. Furthermore, the contents of the various NMDSs are described to very different extents in the articles, which makes it difficult to compare them with each other. In particular, an original dataset could be found for only 1 NMDS (the United States). The articles are also all from different periods, and for some NMDSs, it is unclear whether they are still up to date. There is also no information .

### Comparison With Prior Work

In comparison with previous works, this scoping review provides a broad overview of past and current efforts and initiatives aimed at developing NMDSs in long-term care settings. It offers new insights into the progression of the development and establishment of NMDSs internationally, providing an overview of the content and recommendations for NMDSs in long-term care over the years. The specification of setting distinguishes it from the umbrella review on NMDSs in hospital settings by Freguia et al [2], which focuses on existing NMDSs, their content, and recommendations regarding NMDSs, serving as an orientation for this scoping review. The mapping review by Hanratty et al [14], which was published as a preprint as part of the DACHA study from the United Kingdom, focuses on original research using data from an NMDS in care homes. The study aimed to describe the research applications of data from care home NMDSs and capture key health indicators [14].

In contrast to the reviews noted above, this review focuses generally on NMDSs in the long-term care context, distinguishing it from reviews on hospital settings or research applications. Each type of review provides valuable insights into challenges and approaches across different nursing contexts.

### Conclusions

In this work, the current state of research on NMDSs in long-term care is presented. The concept of NMDSs dates back to the 1990s but has once again become a focus owing to an ever-increasing amount of data and growing digitalization that has resulted in the need for structured and standardized nursing data. The identified NMDSs seem to contain more patient data than nursing data, which is likely related to different understandings and definitions of nursing in different countries. In the future, both this aspect and residents’ needs should be considered in the NMDS development process.

## Supplementary material

10.2196/68670 Multimedia Appendix 1 Database search.

10.2196/68670 Multimedia Appendix 2 Data extraction in MAXQDA.

10.2196/68670 Multimedia Appendix 3 Characteristics of the sources of evidence.

10.2196/68670 Multimedia Appendix 4 Nursing minimum datasets (NMDSs) or initiatives identified for developing NMDSs.

10.2196/68670 Multimedia Appendix 5 Contents of the identified nursing minimum datasets.

10.2196/68670 Multimedia Appendix 6 Recommendations for nursing minimum datasets.

10.2196/68670 Checklist 1 PRISMA-ScR checklist.

## Abbreviations

DACHA: Developing Resources and Minimum Data Set for Care Homes’ Adoption
HI:NC: Health Information: Nursing Components
MDS: minimum dataset
MII: Medical Informatics Initiative
NMDS: nursing minimum dataset
NRS: National Rehabilitation Reporting System
PRISMA: Preferred Reporting Items for Systematic Reviews and Meta-Analyses
PRISMA-ScR: Preferred Reporting Items for Systematic Reviews and Meta-Analyses Extension for Scoping Reviews
