# Supplementary material for: Nursing Minimum Datasets in Long-Term Care Settings: Scoping Review
Source: J Med Internet Res. 2025 Oct 14;27:e68670. doi: 10.2196/68670 (PMC12521810; doi:10.2196/68670)
Supplement: Multimedia Appendix 1 [file jmir-v27-e68670-s001.docx]

# **Appendix 1 – Database search**

| **Search** | **Query** | **Results** |
| --- | --- | --- |
| **PubMed** |  |  |
| #27 | #10 AND #26 | 1981 |
| #26 | #11 OR #12 OR #13 OR #14 OR #15 OR #16 OR #17 OR #18 OR #19 OR #20 OR #21 OR #22 OR #23 OR #24 OR #25 | 467.354 |
| #25 | Rehabilitation"[Mesh] | 320.393 |
| #24 | "Psychiatric Rehabilitation"[Mesh] | 617 |
| #23 | "Nursing homes"[Mesh] | 41.346 |
| #22 | "Homes for the aged"[Mesh] | 13.175 |
| #21 | "Long-term care"[Mesh] | 26.397 |
| #20 | "Rehabilitation care"[Title/Abstract] | 1023 |
| #18 | "Psychiatric facility"[Title/Abstract] | 647 |
| #17 | "Nursing facility"[Title/Abstract] | 3033 |
| #16 | "Care facility"[Title/Abstract] | 9124 |
| #15 | "Homes for the elderly"[Title/Abstract] | 2 |
| #14 | "Nursing home"[Title/Abstract] | 23.937 |
| #13 | "Care home"[Title/Abstract] | 2932 |
| #12 | "Long-term care"[Title/Abstract] | 24.630 |
| #11 | "Longterm care"[Title/Abstract] | 98 |
| #10 | #1 OR #2 OR #3 OR #4 OR #5 OR #6 OR #7 OR #8 OR #9 | 23.759 |
| #9 | NHMDS[Title/Abstract] | 5 |
| #8 | NMDS[Title/Abstract] | 961 |
| #7 | MDS[Title/Abstract] | 19.611 |
| #6 | "Nursing minimum dataset"[Title/Abstract] | 10 |
| #5 | "Nursing minimum data set"[Title/Abstract] | 106 |
| #4 | "Minimum data set"[Title/Abstract] | 2245 |
| #3 | "Minimum dataset"[Title/Abstract] | 494 |
| #2 | “core dataset"[Title/Abstract] | 129 |
| #1 | „core data Set“[Tilte/Abstract] | 198 |
| **CINAHL** |  |  |
| S29 | S12 AND S28 | 1854 |
| S28 | S11 OR S12 OR S13 OR S14 OR S15 OR S16 OR S17 OR S18 OR S19 OR S20 OR S21 OR S22 OR S23 OR S24 OR S25 OR S26 OR S27 | 77.123 |
| S27 | MH "Rehabilitation patients" | 3599 |
| S26 | MH "Rehabilitation centers" | 8681 |
| S25 | MH "Nursing home Patients" | 15.459 |
| S24 | MH "long term care" | 28.629 |
| S23 | TI "Rehabilitation care" OR AB "Rehabilitation care" | 765 |
| S22 | TI "psychiatric facility" OR AB "psychiatric facility" | 295 |
| S21 | TI “Nursing facility” OR AB “Nursing facility” | 2226 |
| S20 | TI "care facility" OR AB "care facility" | 5280 |
| S19 | TI "homes for the elderly" OR AB "homes for the elderly" | 257 |
| S18 | TI "Retirement home" OR AB "Retirement home" | 93 |
| S17 | TI "Nursing home" OR AB "Nursing home" | 18.387 |
| S16 | TI "carehome" OR AB "carehome" | 4 |
| S15 | TI "care home" OR AB "care home" | 3582 |
| S14 | TI "long-term care" OR AB "long-term care" | 19.400 |
| S13 | TI "longterm care" OR AB "longterm care" | 140 |
| S12 | S1 OR S2 OR S3 OR S4 OR S5 OR S6 OR S7 OR S8 OR S9 OR S10 OR S11 | 8469 |
| S11 | MH "nursing Minimum data set" | 428 |
| S10 | MH "Minimum data set" | 2500 |
| S9 | TI NHMDS OR AB NHMDS | 8 |
| S8 | TI NMDS OR AB NMDS | 445 |
| S7 | TI MDS OR AB MDS | 4781 |
| S6 | TI "nursing minimum dataset" OR AB "nursing minimum dataset" | 10 |
| S5 | TI "nursing minimum data set" OR AB "nursing minimum data set" | 119 |
| S4 | TI "minimum data set" OR AB "minimum data set" | 1727 |
| S3 | TI "minimum dataset" OR AB "minimum dataset" | 260 |
| S2 | TI "Core dataset" OR AB "Core dataset" | 39 |
| S1 | TI "Core data set" OR AB "Core data set" | 86 |
| **Embase** |  |  |
| 022 | 10 AND 21 | 2480 |
| 021 | 11 OR 12 OR 13 OR 14 OR 15 OR 16 OR 17 OR 18 OR 19 OR 20 | 6493 |
| 020 | "Rehabilitation care".mp. OR rehabilitation care/ | 428 |
| 019 | "Psychiatric facility".mp. | 28 |
| 018 | "Nursing facility".mp. | 162 |
| 017 | "Care facility".mp. | 2610 |
| 016 | elderly care/ or "Homes for the elderly".mp. OR home for the aged/ | 242 |
| 015 | "Retirement home".mp. OR home for the aged/ | 82 |
| 014 | "Carehome".mp. | 0 |
| 013 | nursing home/ OR "Care home".mp. | 1362 |
| 012 | "Long-term care".mp. OR long term care/ | 2339 |
| 011 | long term care/ OR "Longterm care".mp. | 1998 |
| 010 | 1 OR 2 OR 3 OR 4 OR 5 OR 6 OR 7 OR 8 OR 9 | 5447 |
| 009 | NHMDS.mp. | 0 |
| 008 | NMDS.mp. | 51 |
| 007 | MDS.mp. | 1190 |
| 006 | "Nursing minimum dataset".mp. | 0 |
| 005 | "Nursing minimum data set".mp. OR nursing minimum data set/ | 0 |
| 004 | "Minimum data set".mp. OR sample size/ | 4170 |
| 003 | "Minimum dataset".mp. | 19 |
| 002 | "Core dataset".mp. | 26 |
| 001 | "Core data set".mp. | 12 |
| **DBLP** |  |  |
| 1 | “nursing minimum data set” | 16 |
