# Supplementary material for: Nursing Minimum Datasets in Long-Term Care Settings: Scoping Review
Source: J Med Internet Res. 2025 Oct 14;27:e68670. doi: 10.2196/68670 (PMC12521810; doi:10.2196/68670)
Supplement: Multimedia Appendix 2 [file jmir-v27-e68670-s002.docx]

# **Appendix 2 – Data extraction in Maxqda**

| **Nursing Minimum Data Sets** |
| --- |
| Country specific NMDS |
| Translations/ Transfers of existing NMDS |
| Topic-related NMDS |
| **Contents of the NMDS** |
| Patient Data  - Demographics - Physical and psychosocial factors  - Medical and nursing diagnoses  - Patient’s perception & goal |
| Interpersonal Data  - Nursing Interventions  - Medical & Nursing orders  - Nursing Outcomes |
| Institutional Data |
| **Recommendations** |
| Overall |
| Clinical |
| Research |
| Managerial |
