# Supplementary material for: Nursing Minimum Datasets in Long-Term Care Settings: Scoping Review
Source: J Med Internet Res. 2025 Oct 14;27:e68670. doi: 10.2196/68670 (PMC12521810; doi:10.2196/68670)
Supplement: Multimedia Appendix 3 [file jmir-v27-e68670-s003.docx]

# Appendix 3 – Results

| **Author, Year, Country** | **Title** | **Journal** | **Type** | **Objectives** |
| --- | --- | --- | --- | --- |
| Anliker, M. (2007) Switzerland [34] | Erfahrungen mit RAI in den Alters- und Pflegeheimen der Schweiz | PrInterNet | Overview | To present experiences with the RAI in Switzerland. |
| Anliker M., Bartelt G. (2015) Switzerland [28] | [Resident Assessment Instrument in Switzerland. History, Results and Experiences from the Application] | Zeitschrift für Gerontologie und Geriatrie | Overview | To present adaptations and experiences with the implementation of the Resident Assessment Instrument (RAI) in Swiss long-term care (RAI-HC Schweiz). |
| Armstrong, H., Daly, T., J., Choiniere, J., A. (2017) Canada [31] | Policies and Practices: The Case of RAI-MDS in Canadian Long-Term Care Homes | Journal of Canadian Studies | Review/  Comment | To set the RAI-MDS in the context of neo-liberalism and evidence-based medicine and comment on primary and secondary literature concerning its use. |
| Hanratty, B., Akdur, G., Burton, J.K., Davey, V., Goodman, C., Gordon, A.L., Killett, A., Liddle, J.,  Rand, S., Spilsbury, K., Towers, A. M. (2024) United Kingdom [14] | Application and content of minimum data sets for care homes: A mapping review | medRxiv 2024 | Mapping Review (Preprint) | To describe the research applications of data from care home MDSs, and identify key outcome variables and measures used. |
| Brandenburg, H. (2010) Germany [30] | Das Resident Assessment Instrument (RAI) – Eine Chance für die Pflege in Deutschland |  | Book section | To exemplify the practical relevance of the RAI in Germany. To point out limitations of the RAI. |
| Buhr G., White H. K. (2013) USA [48] | MDS 3.0 Perspective: A Better Tool for Patient Care | Journal of the American Medical Directors Association | Letter to the Editor | To recommend accurate MDS data collection and interpretation in order to improve individual patient care. |
| Burton et al. (2022) United Kingdom [7] | Developing a Minimum Data Set for Older Adult Care Homes in the UK: Exploring the Concept and Defining Early Core Principles | The Lancet Healthy Longevity | Personal View | To set out nine core principles for MDS adoption in nursing homes in the UK. |
| Chen et al. (2018) Taiwan [29] | Applications of Minimum Data Set in Long-term Care Research | Journal of Clinical Gerontology and Geriatrics | Review | To provide an overview of the development of the MDS-LTCF in Taiwan and related research using MDS-LTCF. |
| Clauser S. B., Fries B. E. (1992) USA [32] | Nursing Home Resident Assessment and Case-mix Classification: Cross-national Perspectives | Health Care Financing Review | Review | To explore the potential of RAI and RUGs to aid in cross-national comparative (policy) research. |
| Dellefield M. E., Corazzini K. (2015) USA [25] | Comprehensive Care Plan Development Using Resident Assessment Instrument Framework: Past, Present, and Future Practices | Healthcare | Review | To identify comprehensive care plans (CCPs) developed within the RAI framework in order to give recommendations on CCP development. |
| Dougherty M., Mitchell S. (2004) USA [45] | Getting Better Data from the MDS: Improving Diagnostic Data Reporting in Long-term Care Facilities | Journal of American Health Information Management Association (AHIMA) | Overview | To adjust AHIMA´s recommendations on the quality of MDS 3.0 diagnostic data (Section I). |
| Dupuis M., Fagan F. D. (2003) USA [44] | Understanding the Basics of MDS | Canadian Nursing Home | Overview | To address MDS 2.0 in Q&A format on definition, development, usage and impact. |
| Gilgen R., Garms-Homolova V. (1995) Germany [26] | [The Resident Assessment Instrument: Minimum Data Set and Resident Assessment Protocols - Prerequisites for the implementation in German-speaking countries] | Zeitschrift für Gerontologie und Geriatrie | Discussion Article | To discuss requirements for MDS 1.0 adoption in Germany. |
| Goossen et al. (1998) Netherlands [35] | A Comparison of Nursing Minimal Data Sets | Journal of the American Medical Informatics Association | Discussion Article | To examine the advantages and disadvantages of data sets of nursing practice, and the differences and similarities of five national and international NMDS systems.  To apply this information toward an NMDS initiative in the Netherlands. |
| A. L. Gordon, S. Rand, E. Crellin, S. Allan, F. Tracey, K. De Corte, et al. (2024)  United Kingdom [38] | Piloting a minimum data set for older people living in care homes in England: A developmental study | medRxiv 2024 | Development Study (Preprint) | To assess the feasibility of extracting data directly from DCRs and linking these to routinely collected health and social care data to populate a pilot care home MDS. To assess the quality and completeness of MDS data. |
| Grebe C., Brandenburg H. (2015) Germany [27] | [Resident Assessment Instrument. Application Options and Relevance for Germany] | Zeitschrift für Gerontologie und Geriatrie | Discussion Article | To discuss RAI uses and requirements in German long-term care. |
| A. Killett, K. Micklewright, R. Carroll, G. Akdur, E. Allinson, L. Crellin, et al. (2024)  United Kingdom [12] | Public involvement to enhance care home research; collaboration on a minimum data set for care homes | medRxiv 2024 | Research Article | To present an analysis of the involvement of care home staff and family members of care home residents in the DACHA project. |
| Mallette, C. (2003) Canada [36] | Nursing Minimum Data Sets | Nursing sensitive outcomes: State of nursing science (Book) | Book section / Review | To review nursing-specific databases or nursing minimum data sets. To discuss the classification systems most commonly used in nursing minimum data sets (NMDS). |
| Martin C.M. (2010) USA [46] | Getting ready for MDS 3.0: Patient Evaluation Takes a New Turn | The Consultant Pharmacist | Overview | To present MDS 2.0 issues and to highlight MDS 3.0 improvements based on pharmacists’ perspective. |
| Martin C.M. (2011) USA [47] | MDS: Valuable Clinical Data that Can Help Improve Medication Therapy | The Consultant Pharmacist | Overview | To describe improvements and recommendations using MDS 3.0 for pharmacist medication review. |
| Morley J. E. (2013) USA [50] | Minimum Data Set 3.0: A Giant Step Forward | Journal of the American Medical Directors Association | Editorial | To reflect MDS 3.0 clinical utility in US nursing homes. |
| Ness K. (2000) USA [53] | The Use of the MDS 2.0 to Measure Rehabilitation Outcomes in Skilled Nursing Facilities | Journal of Rehabilitation Outcomes Measurement | Overview | To emphasize MDS 2.0 as a tool for therapists to measure functional outcomes in skilled nursing facilities. |
| Rahman A. N., Applebaum R. A. (2009) USA [52] | The Nursing Home Minimum Data Set Assessment Instrument: Manifest Functions and Unintended Consequences - Past, Present, and Future | Gerontologist | Overview | To assess MDS 3.0 strengths and weaknesses by comparison to MDS 1.0 goals. |
| Rantz M. J., Zwygart-Stauffacher M. & Wipke-Tevis D. (1999) USA [5] | Minimum Data Set and Resident Assessment Instrument: Can Using Standardized Assessment Improve Clinical Practice and Outcomes of Care? | Journal of Gerontological Nursing | Overview | To provide historical information on MDS, describe the importance of standardized assessment and suggest the MDS/RAI be viewed as a pivotal aspect to improving care  and care outcomes. |
| Ryan J., Stone R. I. & Raynor C. R. (2004) USA [24] | Using Large Data Sets in Long-term Care to Measure and Improve Quality | Nursing Outlook | Overview | To address the evolution, potential uses and limitations of large data sets in long-term  care (LTC).  To promote research on the design, implementation and monitoring of quality improvement efforts in nursing homes. |
| Saliba et al. (2012) USA [23] | Overview of Significant Changes in the Minimum Data Set for Nursing Homes Version 3.0 | Journal of the American Medical Directors Association | Discussion article | To highlight and reflect significant clinical changes in MDS 3.0 |
| Salva A., Becker C. (2007) [41] Comprehensive | Minimum Data Set for Research Studies in Falls and Osteoporosis | Journal of Nutrition, Health & Aging | Overview | To provide definitions, methodology and identification of core outcome measures for research studies on falls and osteoporosis. |
| Salva et al. (2004) [40] Comprehensive | Minimum Data Set for Nutritional Intervention Studies in the Elderly IAG/IANA Task Force Consensus | Journal of Nutrition, Health & Aging | Overview | To develop a minimum data set for intervention studies on nutrition. |
| Sinclair A. J. (2007) [42] Comprehensive | Towards a Minimum Data Set for Intervention Studies in Type 2 Diabetes in Older People | Journal of Nutrition, Health & Aging | Overview | To develop a minimum data set for intervention studies in type 2 diabetes in older people. |
| Stewart K., Worden A., Challis D. (2003) United Kingdom [33] | Assessing the Needs of Older People in Care Homes | Nursing & Residential Care | Clinical Review | To analyze the potential of MDS/RAI for standardized assessment in care homes in the UK. |
| Tangalos E. G. (2012) USA [49] | MDS 3.0: Can This Release Be All Things to All People? | Journal of the American Medical Directors Association | Editorial | To promote MDS 3.0 for its clinical use and its use in research. |
| Towers A-M, Gordon A, Wolters AT, et al (2023) United Kingdom [39] | Piloting of a minimum data set for older people living in care homes in England: protocol for a longitudinal, mixed-methods study. | BMJ Open | Mixed-Methods Study | To assess the feasibility of extracting data directly from digital care records and matching this to routinely collected health and social care data to populate a complete MDS. To assess the quality of the complete MDS data.  To evaluate the usefulness of the MDS to stakeholders (health and social care, local authorities, care providers, residents and their families).  To assess potential barriers and facilitators to wider implementation of the MDS. |
| Wells et al. (2009) Canada [37] | Uses of the National Rehabilitation Reporting System: Perspectives of Geriatric Rehabilitation Clinicians | Canadian Journal of Occupational Therapy | Overview | To explore current and potential clinical uses of National Rehabilitation Reporting System (NRS) data. |
| Werley, H. H. et al. (1991) USA [1] | The Nursing Minimum Data Set: Abstraction Tool for Standardized, Comparable, Essential Data. | American Journal of Public Health | Discussion Article | To discuss briefly the following aspects of the NMDS: background including definition, purposes, and elements; availability and reliability of the data; benefits; implications for the NMDS with emphasis on nursing research; and health policy decision making. |
| Zimmermann et al. (2012) USA [51] | Psychosocial Care in Nursing Homes in the Era of the MDS 3.0: Perspectives of the Experts | Journal of Gerontological Social Work | Overview | To report MDS 3.0 recommendations on psychosocial care in nursing homes. |
| Centers for Medicare & Medicaid Services (CMS) (2023) USA [43] | Minimum Data Set (MDS) 3.0 Resident Assessment Instrument (RAI) Manual | U.S. Department of Health & Human Services | Dataset |  |

**References**

1. Werley HH, Devine EC, Zorn CR, Ryan P, Westra BL. The nursing minimum data set: abstraction tool for standardized, comparable, essential data. Am J Public Health. Apr 1991;81(4):421-426. [doi: 10.2105/ajph.81.4.421] [Medline: 2003618]

5. Rantz MJ, Popejoy L, Zwygart-Stauffacher M, Wipke-Tevis D, Grando VT. Minimum data set and resident assessment instrument. Can using standardized assessment improve clinical practice and outcomes of care? J Gerontol Nurs. Jun 1999;25(6):35-43. [doi: 10.3928/0098-9134-19990601-08] [Medline: 10603812]

7. Burton JK, Wolters AT, Towers AM, et al. Developing a minimum data set for older adult care homes in the UK: exploring the concept and defining early core principles. Lancet Healthy Longev. Mar 2022;3(3):e186-e193. [doi: 10.1016/S2666-7568(22)00010-1] [Medline: 35282598]

12. Killett A, Micklewright K, Carroll R, et al. Public involvement to enhance care home research; collaboration on a minimum data set for care homes. Health Expect. Feb 2025;28(1):e70140. [doi: 10.1111/hex.70140] [Medline: 39806859]

14. Hanratty B, Akdur G, Burton JK. Application and content of minimum data sets for care homes: a mapping review. medRxiv. Preprint posted online on Jun 24, 2024. URL: <https://www.medrxiv.org/content/10.1101/2024.06.24.24309361v1> [doi: 10.1101/2024.06.24.24309361v1]

23. Saliba D, Jones M, Streim J, Ouslander J, Berlowitz D, Buchanan J. Overview of significant changes in the minimum data set for nursing homes version 3.0. J Am Med Dir Assoc. Sep 2012;13(7):595-601. [doi: 10.1016/j.jamda.2012.06.001] [Medline: 22784698]

24. Ryan J, Stone RI, Raynor CR. Using large data sets in long-term care to measure and improve quality. Nurs Outlook. 2004;52(1):38-44. [doi: 10.1016/j.outlook.2003.11.001] [Medline: 15014378]

25. Dellefield ME, Corazzini K. Comprehensive care plan development using resident assessment instrument framework: past, present, and future practices. Healthcare (Basel). Oct 26, 2015;3(4):1031-1053. [doi: 10.3390/healthcare3041031] [Medline: 27417811]

26. Gilgen R, Garms-Homolova V. The Resident Assessment Instrument: minimum data set and resident assessment protocols - prerequisites for the implementation in German-speaking countries. [German]. Resident Assessment Instrument (RAI): System zur klientenbeurteilung und dokumentation in der langzeitpflege - eine ubersicht. Short Survey Zeitschrift fur Gerontologie. 1995;28(1):25-28. [Medline: 7773827]

27. Grebe C, Brandenburg H. Resident assessment instrument. Application options and relevance for Germany. Z Gerontol Geriatr. Feb 2015;48(2):105-113. [doi: 10.1007/s00391-015-0855-6] [Medline: 25676014]

28. Anliker M, Bartelt G. Resident assessment instrument in Switzerland. History, results and experiences from the application. Z Gerontol Geriatr. Feb 2015;48(2):114-120. [doi: 10.1007/s00391-015-0864-5] [Medline: 25676015]

29. Chen LY, Lin MH, Peng LN, Chen LK. Applications of minimum data set in long-term care research. Aging Med Healthc. 2018;9(4):118-125. URL: <https://www.e-jcgg.com/?p=6339> [doi: 10.33879/JCGG.2018.1811]

30. Brandenburg H. Das Resident Assessment Instrument (RAI) - eine chance für die pflege in Deutschland. In: Bartholomeyczik S, Halek M, editors. Assessmentintrumente in Der Pflege: Möglichkeiten Und Grenzen. Schlütersche; 2010:27-47. ISBN: 9783899932249

31. Armstrong H, Daly TJ, Choiniere JA. Policies and practices: the case of RAI-MDS in Canadian long-term care homes. JCS. May 2017;50(2):348-367. [doi: 10.3138/jcs.50.2.348]

32. Clauser SB, Fries BE. Nursing home resident assessment and case-mix classification: cross-national perspectives. Health Care Financ Rev. 1992;13(4):135-155. [Medline: 10122002]

33. Stewart K, Worden A, Challis D. Assessing the needs of older people in care homes. Nursing and Residential Care. Jan 2003;5(1):22-25. [doi: 10.12968/nrec.2003.5.1.10962]

34. Anliker M. Experiences with RAI in Suisse geriatric nursing and retirement homes. PR-Internet fur die Pflege. 2007;9(5):332-336.

35. Goossen WT, Epping PJ, Feuth T, Dassen TW, Hasman A, van den Heuvel WJ. A comparison of nursing minimal data sets. J Am Med Inform Assoc. 1998;5(2):152-163. [doi: 10.1136/jamia.1998.0050152] [Medline: 9524348]

36. Mallette C. Nursing Minimum Data Sets Nursing-Sensitive Outcomes. Jones & Bartlett Learning; 2003. ISBN: 9780763722876

37. Wells JL, Egan M, Byrne K, Jaglal S, Dumbrell AC, Stolee P. Uses of the National Rehabilitation Reporting System: perspectives of geriatric rehabilitation clinicians. Can J Occup Ther. Oct 2009;76(4):294-298. [doi: 10.1177/000841740907600408] [Medline: 19891299]

38. Gordon AL, Rand S, Crellin E, et al. Piloting a minimum data set for older people living in care homes in England: a developmental study. Age Ageing. Jan 6, 2025;54(1):afaf001. [doi: 10.1093/ageing/afaf001] [Medline: 39812411]

39. Towers AM, Gordon A, Wolters AT, et al. Piloting of a minimum data set for older people living in care homes in England: protocol for a longitudinal, mixed-methods study. BMJ Open. Feb 27, 2023;13(2):e071686. [doi: 10.1136/bmjopen-2023-071686] [Medline: 36849214]

40. Salva A, Corman B, Andrieu S, et al. Minimum data set for nutritional intervention studies in the elderly IAG/ IANA task force consensus. J Nutr Health Aging. 2004;8(4):202-206. [Medline: 15316582]

41. Salva A, Becker C. Minimum data set for research studies in falls and osteoporosis. Geronto Net. J Nutr Health Aging. 2007;11(3):283-287. [Medline: 17508109]

42. Sinclair AJ. Towards a minimum data set for intervention studies in type 2 diabetes in older people. J Nutr Health Aging. 2007;11(3):289-293. [Medline: 17508110]

43. Minimum data set (MDS) 3.0 resident assessment instrument (RAI) manual. Centers for Medicare & Medicaid Services. 2024. URL: <https://www.cms.gov/medicare/quality/nursing-home-improvement/resident-assessment-instrument-manual> [Accessed 2025-09-02]

44. Dupuis M, Fagan FD. Understanding the basics of MDS. Can Nurs Home. 2003;14(5):5-11.

45. Dougherty M, Mitchell S. Getting better data from the MDS. Improving diagnostic data reporting in long-term care facilities. J AHIMA. 2004;75(10):28-33. [Medline: 15559836]

46. Martin CM. Getting ready for MDS 3.0: patient evaluation takes a new turn. Consult Pharm. Jul 2010;25(7):404-406. [doi: 10.4140/TCP.n.2010.404] [Medline: 20601346]

47. Martin CM. MDS: valuable clinical data that can help improve medication therapy. Consult Pharm. Oct 2011;26(10):710-714. [doi: 10.4140/TCP.n.2011.710] [Medline: 22005138]

48. Buhr G, White HK. MDS 3.0 perspective: a better tool for patient care. J Am Med Dir Assoc. Mar 2013;14(3):221-222. [doi: 10.1016/j.jamda.2012.11.014] [Medline: 23318045]

49. Tangalos EG. MDS 3.0: can this release be all things to all people? J Am Med Dir Assoc. Sep 2012;13(7):576-577. [doi: 10.1016/j.jamda.2012.04.015] [Medline: 22698952]

50. Morley JE. Minimum data set 3.0: a giant step forward. J Am Med Dir Assoc. Jan 2013;14(1):1-3. [doi: 10.1016/j.jamda.2012.10.014] [Medline: 23200806]

51. Zimmerman S, Connolly R, Zlotnik JL, Bern-Klug M, Cohen LW. Psychosocial care in nursing homes in the era of the MDS 3.0: perspectives of the experts. J Gerontol Soc Work. 2012;55(5):444-461. [doi: 10.1080/01634372.2012.667525] [Medline: 22783960]

52. Rahman AN, Applebaum RA. The nursing home minimum data set assessment instrument: manifest functions and unintended consequences--past, present, and future. Gerontologist. Dec 2009;49(6):727-735. [doi: 10.1093/geront/gnp066] [Medline: 19531805]

53. Ness K. The use of the MDS 2.0 to measure rehabilitation outcomes in skilled nursing facilities. JROM. 2000;4(2):22-30.
