# Supplementary material for: Nursing Minimum Datasets in Long-Term Care Settings: Scoping Review
Source: J Med Internet Res. 2025 Oct 14;27:e68670. doi: 10.2196/68670 (PMC12521810; doi:10.2196/68670)
Supplement: Multimedia Appendix 4 [file jmir-v27-e68670-s004.docx]

# Appendix 4 – Results

| Country specific NMDSs | | | | | | |
| --- | --- | --- | --- | --- | --- | --- |
| **Minimum Data Sets** | MDS USA (1.0-3.0) | NMDS-N | Telenurse Project | Health Information: Nursing components (HI:NC) | National Rehabilitation Reporting System (NRS) MDS | MDS UK |
| **Country** | USA | Netherlands | European Countries | Canada | Canada | UK |
| **Source** | e.g., Werley et al., 1991 | Goossen et al. 1998 | Goossen et al. 1998 | Goossen et al. 1998, Mallette 2003 | Wells 2009 | Towers et al. 2023; Burton et al. 2022; Hanratty et al. 2024; Gordon et al. 2024; Killett et al. 2024 |
| **Purpose** | 1. Establish comparability of nursing data across clinical populations, settings, geographic areas, and time; 2. Describe the nursing care of clients and their families in a variety of settings; 3. Demonstrate or project trends regarding nursing care provided and allocation of nursing resources to individuals or populations according to their health problems, or nursing diagnoses; 4. Stimulate nursing research using the NMDS elements alone, as well as through links to the more detailed data existing in nursing and other health care information systems; 5. Provide data about nursing care to influence and facilitate health policy decision making. [1] | e.g., budgeting, determining the effectiveness of care, presenting epidemiologic data on nursing problems, and supporting policy making. [35] | 1. Determine feasibility of nursing data collection and comparison in Europe; 2. Make visible what nurses do Collect nursing data that have been documented with use of the ICNP [35]; 3. description of the diversity of patient populations from a nursing point of view (nursing diagnoses/problems); 4. Description of the variability of practice patterns (nursing interventions); 5. description of the clinical results of nursing care (outcomes) on a European level. [35] | 1. Deliver information about nursing care; 2. Demonstrate unique contribution of nurses to the health of Canadians. [35] | 1. Facilitate the collection of information on service utilization and outcomes from inpatient rehabilitation services across Canada; 2. Support management decision making at facility, regional, provincial/territorial, and national levels; 3. Allow provincial/territorial and national comparative reporting. [37] | 1. Improving the accessibility and interoperability of routinely collected data in care homes; 2. Standardizing the way in which data on residents' needs and events is collected. [39] |
| **Translations** | Czech, Danish, Dutch, French,[25] German, [25-27] Icelandic, Italian, Norwegian, Spanish, Swedish,[25] Swiss,[28] Taiwan [29] | - | - | - | - | - |
| **Status** | Active; first Version (1.0) implemented in 1990; Last version (3.0) first implemented in 2015. | Status 1998: ongoing project. [35] | Status 1998: ongoing project. [35] | Status 1998: ongoing project. [35] | Status 2009: Since 2003, use of the NRS has been mandated in all Ontario inpatient adult rehabilitation facilities, including eight specialized units providing geriatric rehabilitation. [37] | Under development; Part of DACHA study (2019-2024); There have been previous attempts to implement an MDS UK that were not successful. [7,33] |
|  |  |  |  |  |  |  |
| Minimum Data Sets for Research / Topic related MDS | | | | | | |
| **Minimum Data Set** | MDS for nutritional interventions studies in the elderly | MDS for research studies in falls and osteoporosis \| Core data set for falls recommended by ProFaNe | MDS for intervention studies in type 2 diabetes in older adults |  |  |  |
| **Topic** | Nutrition \| Intervention studies | Falls \| Osteoporosis \| Research studies | Type 2 Diabetes \| Intervention studies |  |  |  |
| **Source** | Salva et al. 2004 | Salva & Becker, 2007 | Sinclair 2007 |  |  |  |
| **Purpose** | Overcome the lack of standardization in protocols and outcomes in nutritional interventions in the elderly.[40] | Standardization of methods and interventions for the topics of falls and osteoporosis. [41] | 1. Provide a standardized method of assessment and outcome measures for conducting large scale intervention studies with a randomized controlled design; 2. Enable valid comparisons of research findings in different populations of patients; 3. Allow a more detailed analysis of the validity, reliability and sensitivity of existing measures and promote the development of new measures suitable for studies in older People; 4. By systematic review procedures and meta-analyses of studies using a recognized minimum data set, there will be an increased likelihood of demonstrating both clinical and cost-effectiveness of a range of interventions. [42] |  |  |  |
| **Status** | Status 2004: Content development completed.[40] | Status 2007: Content of the core data set for falls recommended by the ProFaNe is used.[41] | Status 2007: Contents are defined and recommended.[42] |  |  |  |

**References**

1. Werley HH, Devine EC, Zorn CR, Ryan P, Westra BL. The nursing minimum data set: abstraction tool for standardized, comparable, essential data. Am J Public Health. Apr 1991;81(4):421-426. [doi: 10.2105/ajph.81.4.421] [Medline: 2003618]

7. Burton JK, Wolters AT, Towers AM, et al. Developing a minimum data set for older adult care homes in the UK: exploring the concept and defining early core principles. Lancet Healthy Longev. Mar 2022;3(3):e186-e193. [doi: 10.1016/S2666-7568(22)00010-1] [Medline: 35282598]

24. Ryan J, Stone RI, Raynor CR. Using large data sets in long-term care to measure and improve quality. Nurs Outlook. 2004;52(1):38-44. [doi: 10.1016/j.outlook.2003.11.001] [Medline: 15014378]

25. Dellefield ME, Corazzini K. Comprehensive care plan development using resident assessment instrument framework: past, present, and future practices. Healthcare (Basel). Oct 26, 2015;3(4):1031-1053. [doi: 10.3390/healthcare3041031] [Medline: 27417811]

26. Gilgen R, Garms-Homolova V. The Resident Assessment Instrument: minimum data set and resident assessment protocols - prerequisites for the implementation in German-speaking countries. [German]. Resident Assessment Instrument (RAI): System zur klientenbeurteilung und dokumentation in der langzeitpflege - eine ubersicht. Short Survey Zeitschrift fur Gerontologie. 1995;28(1):25-28. [Medline: 7773827]

27. Grebe C, Brandenburg H. Resident assessment instrument. Application options and relevance for Germany. Z Gerontol Geriatr. Feb 2015;48(2):105-113. [doi: 10.1007/s00391-015-0855-6] [Medline: 25676014]

28. Anliker M, Bartelt G. Resident assessment instrument in Switzerland. History, results and experiences from the application. Z Gerontol Geriatr. Feb 2015;48(2):114-120. [doi: 10.1007/s00391-015-0864-5] [Medline: 25676015]

33. Stewart K, Worden A, Challis D. Assessing the needs of older people in care homes. Nursing and Residential Care. Jan 2003;5(1):22-25. [doi: 10.12968/nrec.2003.5.1.10962]

35. Goossen WT, Epping PJ, Feuth T, Dassen TW, Hasman A, van den Heuvel WJ. A comparison of nursing minimal data sets. J Am Med Inform Assoc. 1998;5(2):152-163. [doi: 10.1136/jamia.1998.0050152] [Medline: 9524348]

36. Mallette C. Nursing Minimum Data Sets Nursing-Sensitive Outcomes. Jones & Bartlett Learning; 2003. ISBN: 9780763722876

37. Wells JL, Egan M, Byrne K, Jaglal S, Dumbrell AC, Stolee P. Uses of the National Rehabilitation Reporting System: perspectives of geriatric rehabilitation clinicians. Can J Occup Ther. Oct 2009;76(4):294-298. [doi: 10.1177/000841740907600408] [Medline: 19891299]

39. Towers AM, Gordon A, Wolters AT, et al. Piloting of a minimum data set for older people living in care homes in England: protocol for a longitudinal, mixed-methods study. BMJ Open. Feb 27, 2023;13(2):e071686. [doi: 10.1136/bmjopen-2023-071686] [Medline: 36849214]

40. Salva A, Corman B, Andrieu S, et al. Minimum data set for nutritional intervention studies in the elderly IAG/ IANA task force consensus. J Nutr Health Aging. 2004;8(4):202-206. [Medline: 15316582]

41. Salva A, Becker C. Minimum data set for research studies in falls and osteoporosis. Geronto Net. J Nutr Health Aging. 2007;11(3):283-287. [Medline: 17508109]

42. Sinclair AJ. Towards a minimum data set for intervention studies in type 2 diabetes in older people. J Nutr Health Aging. 2007;11(3):289-293. [Medline: 17508110]
