# Supplementary material for: Nursing Minimum Datasets in Long-Term Care Settings: Scoping Review
Source: J Med Internet Res. 2025 Oct 14;27:e68670. doi: 10.2196/68670 (PMC12521810; doi:10.2196/68670)
Supplement: Multimedia Appendix 5 [file jmir-v27-e68670-s005.docx]

# Appendix 5 – Results

|  | **Patient Data** | | | **Interpersonal Data** | | | **Institutional Data** |
| --- | --- | --- | --- | --- | --- | --- | --- |
| **Minimum Data Set** | **Demographics** | **Physiological & Psychosocial factors and Diagnoses** | **Patients’ Perception & Goals** | **Interventions** | **Nursing & Medical Orders** | **Goal (Outcome)** |  |
| **MDS USA** | Identification Information Demographics [1,23,35,43,44] | Active Diagnoses [1,23,26,43-45] - Cancer [44] - Heart/Circulation [43] - Gastrointestinal [43] - Infections [43,45] - Metabolic [43] - Musculoskeletal [43] - Neurological [43] - Nutritional [34] - Psychiatric/Mood Disorder [23,27,29,43,46-50,52] - Pulmonary [43] - Vision [43] | Preferences for customary routine and activities [27,43,50,52] | Special treatments, procedures & programs [26,43,44] | Restrains & Alarms [27,43] |  | Assessment Information [5,43,44] Health record number of patient/residents [1,26,44] [43] Facility Information [1] Episodic Information [43,44] |
|  |  | Health Condition [1,27,43] - Pain [27,43,46,48,50,51] - Fall [23,43,46,50] - Dyspnea [43] | Functional ability and goals [44] - Everyday Activities [23,26,27,29,43,44,53] - Mobility [43,53] - Self-Care [43] | Psychosocial Care [45] | Catheter [23] |  |  |
|  |  | Hearing, Speech & Vision [26,27,43,44] | Participation in Assessment & Goal setting [43] |  | Medication [1,26,28,43,44,46] |  |  |
|  |  | Cognitive Patterns [23,26,27,29,43,44,47-49,51,52] | Quality of Life [52] |  |  |  |  |
|  |  | Mood [43] | Habits [27] |  |  |  |  |
|  |  | Behavior [26,27,29,43,45,46] |  |  |  |  |  |
|  |  | Bladder and Bowel [23,26,27,43,44,52] |  |  |  |  |  |
|  |  | Swallowing/Nutritional Status [27,43,45] |  |  |  |  |  |
|  |  | Oral/dental status [26,27,43,44] |  |  |  |  |  |
|  |  | Skin Conditions [23,26,27,43,44,52] |  |  |  |  |  |
| **NMDS-N** | - | - |  |  |  |  |  |
| **NRS** | Client demographics [37] | Functional status [37] |  |  |  |  |  |
| **Telenurse Project** | Patient demographics [35] | Nursing diagnosis [35] |  | Nursing Intervention [35] |  | Nursing Outcome [35] | Type of institution [35] |
|  |  |  |  |  |  |  | Moment of stay (admission, stay, discharge [35] |
| **HI:NC** | Patient demographics [35] | Medical diagnoses [35] |  | Procedures [35] |  | Client Outcomes [35] | Facility Information [35] |
|  |  |  |  | Nursing Intervention [35] |  |  | Staff Information [35] |
|  |  |  |  | Nursing Intensity [35] |  |  |  |
| **MDS UK** | Demographics/Characteristics [39] | Diagnoses [39] - Medical History,  - Frailty,  - Adverse Reactions and Allergies | Residents Needs [39] Understanding and Need for Support [39]  Quality of life/dementia quality of life [39] | Healthcare Utilization [39] - Primary Care Use,  - Community nursing,  - Out-of-hours contacts - Ambulance call-outs - Accident and Emergency attendance | Medication [39] | Quality of Life Outcomes [39] | Care Home characteristics [39] |
|  | Palliative Care Needs [39] | Resident Needs [39] - Skin Condition - Cognitive impairment and impact on perception, understanding and need for support - Oral/nutritional status, - Continence,  - Ability to perform activities of daily living, - Cognitive performance, - Delirium |  | Complication/adverse events [39] | Vaccination [39] | Applications/adverse events [39] - Infections,  - Falls | Workforce Characteristics [39] |
|  |  | Quality of life/Dementia Quality of Life [39] |  |  |  |  | Care Home Stay [39] |
|  |  | Mood [39] |  |  |  |  |  |
| **MDS Nutrition** | Birth date [40] | Comorbidity [40] |  |  | Medication [40] |  | Place of residence [40] |
|  | Gender [40] | Height [40] |  |  |  |  |  |
|  | Social status [40] | Body Weight [40] |  |  |  |  |  |
|  | Income [40] | BMI [40] |  |  |  |  |  |
|  | Education [40] | Nutrition [40] |  |  |  |  |  |
| **MDS Fall** | Sociodemographic Data [41] | Comorbidity [41] |  |  | Medication [41] | Information Related to Falls [41] |  |
|  |  | Gait and Balance [41] |  |  |  | Injuries (ICD 10) [41] |  |
|  |  | Mental Status [41] |  |  |  | Physical Activity [41] |  |
|  |  | Nutrition [41] |  |  |  | Activities of daily living [41] |  |
|  |  |  |  |  |  | Health related quality of life [41] |  |
|  |  |  |  |  |  | Psychological Consequences [41] |  |
|  |  |  |  |  |  | Cost-effectiveness measures [41] |  |
| **MDS Diabetes** |  | Laboratory/metabolic parameters [42] |  |  | Medication [42] | Patient centered outcomes [42] |  |
|  |  | Any diabetes-related end-point including erectile dysfunction [42] |  |  |  | Cardiovascular event rate [42] Stroke Rate [42] |  |

**References**

1. Werley HH, Devine EC, Zorn CR, Ryan P, Westra BL. The nursing minimum data set: abstraction tool for standardized, comparable, essential data. Am J Public Health. Apr 1991;81(4):421-426. [doi: 10.2105/ajph.81.4.421] [Medline: 2003618]

5. Rantz MJ, Popejoy L, Zwygart-Stauffacher M, Wipke-Tevis D, Grando VT. Minimum data set and resident assessment instrument. Can using standardized assessment improve clinical practice and outcomes of care? J Gerontol Nurs. Jun 1999;25(6):35-43. [doi: 10.3928/0098-9134-19990601-08] [Medline: 10603812]

23. Saliba D, Jones M, Streim J, Ouslander J, Berlowitz D, Buchanan J. Overview of significant changes in the minimum data set for nursing homes version 3.0. J Am Med Dir Assoc. Sep 2012;13(7):595-601. [doi: 10.1016/j.jamda.2012.06.001] [Medline: 22784698]

26. Gilgen R, Garms-Homolova V. The Resident Assessment Instrument: minimum data set and resident assessment protocols - prerequisites for the implementation in German-speaking countries. [German]. Resident Assessment Instrument (RAI): System zur klientenbeurteilung und dokumentation in der langzeitpflege - eine ubersicht. Short Survey Zeitschrift fur Gerontologie. 1995;28(1):25-28. [Medline: 7773827]

27. Grebe C, Brandenburg H. Resident assessment instrument. Application options and relevance for Germany. Z Gerontol Geriatr. Feb 2015;48(2):105-113. [doi: 10.1007/s00391-015-0855-6] [Medline: 25676014]

28. Anliker M, Bartelt G. Resident assessment instrument in Switzerland. History, results and experiences from the application. Z Gerontol Geriatr. Feb 2015;48(2):114-120. [doi: 10.1007/s00391-015-0864-5] [Medline: 25676015]

29. Chen LY, Lin MH, Peng LN, Chen LK. Applications of minimum data set in long-term care research. Aging Med Healthc. 2018;9(4):118-125. URL: <https://www.e-jcgg.com/?p=6339> [doi: 10.33879/JCGG.2018.1811]

34. Anliker M. Experiences with RAI in Suisse geriatric nursing and retirement homes. PR-Internet fur die Pflege. 2007;9(5):332-336.

35. Goossen WT, Epping PJ, Feuth T, Dassen TW, Hasman A, van den Heuvel WJ. A comparison of nursing minimal data sets. J Am Med Inform Assoc. 1998;5(2):152-163. [doi: 10.1136/jamia.1998.0050152] [Medline: 9524348]

37. Wells JL, Egan M, Byrne K, Jaglal S, Dumbrell AC, Stolee P. Uses of the National Rehabilitation Reporting System: perspectives of geriatric rehabilitation clinicians. Can J Occup Ther. Oct 2009;76(4):294-298. [doi: 10.1177/000841740907600408] [Medline: 19891299]

39. Towers AM, Gordon A, Wolters AT, et al. Piloting of a minimum data set for older people living in care homes in England: protocol for a longitudinal, mixed-methods study. BMJ Open. Feb 27, 2023;13(2):e071686. [doi: 10.1136/bmjopen-2023-071686] [Medline: 36849214]

40. Salva A, Corman B, Andrieu S, et al. Minimum data set for nutritional intervention studies in the elderly IAG/ IANA task force consensus. J Nutr Health Aging. 2004;8(4):202-206. [Medline: 15316582]

41. Salva A, Becker C. Minimum data set for research studies in falls and osteoporosis. Geronto Net. J Nutr Health Aging. 2007;11(3):283-287. [Medline: 17508109]

42. Sinclair AJ. Towards a minimum data set for intervention studies in type 2 diabetes in older people. J Nutr Health Aging. 2007;11(3):289-293. [Medline: 17508110]

43. Minimum data set (MDS) 3.0 resident assessment instrument (RAI) manual. Centers for Medicare & Medicaid Services. 2024. URL: <https://www.cms.gov/medicare/quality/nursing-home-improvement/resident-assessment-instrument-manual> [Accessed 2025-09-02]

44. Dupuis M, Fagan FD. Understanding the basics of MDS. Can Nurs Home. 2003;14(5):5-11.

45. Dougherty M, Mitchell S. Getting better data from the MDS. Improving diagnostic data reporting in long-term care facilities. J AHIMA. 2004;75(10):28-33. [Medline: 15559836]

46. Martin CM. Getting ready for MDS 3.0: patient evaluation takes a new turn. Consult Pharm. Jul 2010;25(7):404-406. [doi: 10.4140/TCP.n.2010.404] [Medline: 20601346]

47. Martin CM. MDS: valuable clinical data that can help improve medication therapy. Consult Pharm. Oct 2011;26(10):710-714. [doi: 10.4140/TCP.n.2011.710] [Medline: 22005138]

48. Buhr G, White HK. MDS 3.0 perspective: a better tool for patient care. J Am Med Dir Assoc. Mar 2013;14(3):221-222. [doi: 10.1016/j.jamda.2012.11.014] [Medline: 23318045]

49. Tangalos EG. MDS 3.0: can this release be all things to all people? J Am Med Dir Assoc. Sep 2012;13(7):576-577. [doi: 10.1016/j.jamda.2012.04.015] [Medline: 22698952]

50. Morley JE. Minimum data set 3.0: a giant step forward. J Am Med Dir Assoc. Jan 2013;14(1):1-3. [doi: 10.1016/j.jamda.2012.10.014] [Medline: 23200806]

51. Zimmerman S, Connolly R, Zlotnik JL, Bern-Klug M, Cohen LW. Psychosocial care in nursing homes in the era of the MDS 3.0: perspectives of the experts. J Gerontol Soc Work. 2012;55(5):444-461. [doi: 10.1080/01634372.2012.667525] [Medline: 22783960]

52. Rahman AN, Applebaum RA. The nursing home minimum data set assessment instrument: manifest functions and unintended consequences--past, present, and future. Gerontologist. Dec 2009;49(6):727-735. [doi: 10.1093/geront/gnp066] [Medline: 19531805]

53. Ness K. The use of the MDS 2.0 to measure rehabilitation outcomes in skilled nursing facilities. JROM. 2000;4(2):22-30.
