# Supplementary material for: Nursing Minimum Datasets in Long-Term Care Settings: Scoping Review
Source: J Med Internet Res. 2025 Oct 14;27:e68670. doi: 10.2196/68670 (PMC12521810; doi:10.2196/68670)
Supplement: Multimedia Appendix 6 [file jmir-v27-e68670-s006.docx]

# Appendix 6 – Results

| **Minimum Data  Set** | **Recommendations** | | | |
| --- | --- | --- | --- | --- |
|  | **Overall** | **Clinical** | **Research** | **Managerial** |
| **NMDS USA** | How to improve accuracy in MDS Section I (Active Diagnoses): A process must be in place to resolve diagnoses and update the clinical information system if it feeds the MDS. Only active diagnoses and those that affect the plan of care should be reported on the MDS. [45] Facilities should have policies in place regarding collecting diagnoses, how they are coded, how they are reported on the MDS, and by whom. [45] New code books or updates should be purchased each year and available to staff who complete section I of the MDS. [45] | Clinicians should be informed of the results of MDS assessments, especially if they reveal problems that have not already been addressed in the medical record. [48] |  | Attending physicians and medical directors should work with nursing home leaders to develop mechanisms to ensure that they are informed of the results of mood, cognitive, delirium and pain assessments on their patients. [48] |
|  | Careful documentation of the care provided and the resources used is particularly relevant, as the NMDS can be used as an abstraction tool for the collection of standardized, comparable data. [1] | The MDS 3.0 contains helpful, quantitative information in the patient's voice. Making full use of the information in the patient’s voice from the MDS should entail enlisting the clinician in the response to cognitive dysfunction, delirium, depression, and pain. [48] |  | Medical directors should help their facility leaders to develop formal protocols for communicating the MDS data to providers. This communication should entail nurses, pharmacists, social workers, therapists, and physicians sharing their interpretation of these data. [48] |
|  | Nursing data must be readily retrievable and available for health policy making. [1] | Careful review of current medications for adverse effects, assessment/diagnosis of acute illness, medical management of pain and depression must continue to the fullest extent to optimize function and quality of life. [48] |  | Nurse administrators should highlight both the need to abstract core, minimum nursing data in public health agencies and the need for these data to be comparable across the various types of care delivery settings, so that they can be incorporated into further development of computerized nursing information systems. [1] |
|  |  | How to improve accuracy in MDS Section I (Active Diagnoses): For improving quality, all reported diagnoses must be supported by physician documentation in the medical record. [46] Individuals who complete section I should be trained in ICD-9-CM coding, with emphasis placed on general and long-term care specific coding rules. [45] |  | Nurse administrators should recognize the trends these core data can reflect about both public health nursing practice and the resources used in providing services, as well as the research possibilities of these data. [1] |
|  |  | The “MDS coordinators” need to become active members of the care team. Physicians need to be informed when abnormalities are present on the admission RAI or on the quarterly assessments. Treatments need to be tailored to the findings of the MDS 3.0. [50] |  |  |
|  |  | MDS training programs that provide hands-on practice with the instrument, (especially with items requiring resident interview or standardized assessment), and timely performance feedback are needed to help assessment nurses adapt to the new protocols and move away from past practices. [52] |  |  |
|  |  | Staff should take care when coding MDS, as the data will be used for many purposes, not least for assessment, payment, regulation and quality improvement. [5] |  |  |
|  |  | Staff need to be supported in gaining the confidence and ability to conduct structured interviews with their residents. [23] |  |  |
|  |  | Both nurses and care managers are responsible for implementing the NMDS elements. Nurses should attach importance to complete and correct documentation of nursing care in accordance with the nursing process. [1] |  |  |
|  |  | Nurse educators must create an awareness in students of the necessity to document care appropriately, reflecting use of the nursing process. [1] |  |  |
|  |  | Faculty should ensure the integration of information management and computerization into undergraduate and graduate curricula for use in decision making in all areas of nursing. [1] |  |  |
|  |  | Nurse educators in undergraduate and graduate programs should stress the relevance of the NMDS as an integral component of nursing information systems. [1] |  |  |
|  |  | Continuing education for public health nurses and clinicians should include basic information regarding the NMDS elements, their definitions, benefits, and implications. [1] |  |  |
|  |  | Nurses in all functional areas can and should find a role for themselves in promoting, implementing, and testing the NMDS in the interest of improving client health care. [1] |  |  |
|  |  | Nurses must be knowledgeable not only about entering data but also about retrieving them, in order to assure the integrity of nursing's essential data. [1] |  |  |
| **MDS UK** | The MDS must primarily focus on measuring what matters most to support those living in care homes through systematic data collection and sharing. [7] |  | It is important to leverage practical knowledge by bridging the gap between research and practice, enabling a dynamic collaboration that can swiftly advance the integration of research insights and professional expertise in social care. [14] |  |
|  | The MDS must reduce data burden and duplication of effort for the care home. This will be achieved through piloting, collaboration, and ongoing engagement with homes. [7] |  | MDS-based researchers should ensure that their questions reflect the priorities of residents and their caregivers, as this will improve data entry and allow the information to be used as a basis for care planning and review. [14] |  |
|  | The MDS must be evidence-based in design and contents, requiring coproduction with key stakeholders. [7] |  |  |  |
|  | MDS data should characterize the population and their needs, including systematically capturing what matters to support those living in care homes and those who support them. [7] |  |  |  |
|  | The MDS should bring together data from within the care home, coupled with data held externally about residents and care services. [7] |  |  |  |
|  | Data sharing with external users of the MDS must have an agreed purpose.  Data sharing pathways must be defined and formalized in data sharing agreements, using secure environments for access where appropriate. [7] Care home residents’ privacy rights must be protected. [7] |  |  |  |
|  | MDS data should be the basis for exploration of variations in care moving beyond raw comparisons or counts to generate evidence from practice, supporting practice development and evaluation of innovation. [7] |  |  |  |
|  | Next steps of development and implementation of NMDS should build on collaborative and communicative relationships between researchers and the public, incorporating relevant knowledge and experience to minimize negative consequences. [12] |  |  |  |
|  | There are key gaps in MDS research, as quality of life (QoL) is often focused on health and functioning instead of broader social care outcomes, that should be addressed. [14] |  |  |  |
| **NMDS N** | Data items need to be identified as variables. Each variable needs to be defined accurately. [35] | Nursing professionals still need to agree on all the care elements and terminology used in this multidisciplinary data set. We recommend that the multidisciplinary approach be investigated further. [35] | For an international comparison, not only the instrument for data collection (i.e., the NMDS) needs to be consistent but also the research questions, sampling methods and sizes, modes of data collection, and means of data analysis must be compatible. [35] |  |
|  | The universe of possible values for each variable or data item must be determined. [35] | Architects of an NMDS must create mechanisms that address informed consent issues and take measures to protect privacy. [35] |  |  |
|  | Nursing data must become available in large multidisciplinary databases to support health care management and policy decisions. [35] |  |  |  |
| **MDS Nutrition** | Compliance should be assessed with great care, as the intention for caregivers to treat. [40] |  |  |  |
| **NMDS Canada (NRS)** |  | It would be very useful to consider clinicians’ perspectives of actual and potential educational and policy uses of NRS data. [37] | Research is required to identify the specific additional data that would make the existing NRS data set more meaningful to geriatric populations. Toolkits of geriatric rehabilitation outcome measures may prove to be a helpful starting point for these investigations. [37] |  |
|  |  |  | Further work would be needed to determine if the costs of customization and the time required for additional data collection would be justified. [37] |  |

**References**

1. Werley HH, Devine EC, Zorn CR, Ryan P, Westra BL. The nursing minimum data set: abstraction tool for standardized, comparable, essential data. Am J Public Health. Apr 1991;81(4):421-426. [doi: 10.2105/ajph.81.4.421] [Medline: 2003618]

7. Burton JK, Wolters AT, Towers AM, et al. Developing a minimum data set for older adult care homes in the UK: exploring the concept and defining early core principles. Lancet Healthy Longev. Mar 2022;3(3):e186-e193. [doi: 10.1016/S2666-7568(22)00010-1] [Medline: 35282598]

12. Killett A, Micklewright K, Carroll R, et al. Public involvement to enhance care home research; collaboration on a minimum data set for care homes. Health Expect. Feb 2025;28(1):e70140. [doi: 10.1111/hex.70140] [Medline: 39806859]

14. Hanratty B, Akdur G, Burton JK. Application and content of minimum data sets for care homes: a mapping review. medRxiv. Preprint posted online on Jun 24, 2024. URL: <https://www.medrxiv.org/content/10.1101/2024.06.24.24309361v1> [doi: 10.1101/2024.06.24.24309361v1]

23. Saliba D, Jones M, Streim J, Ouslander J, Berlowitz D, Buchanan J. Overview of significant changes in the minimum data set for nursing homes version 3.0. J Am Med Dir Assoc. Sep 2012;13(7):595-601. [doi: 10.1016/j.jamda.2012.06.001] [Medline: 22784698]

35. Goossen WT, Epping PJ, Feuth T, Dassen TW, Hasman A, van den Heuvel WJ. A comparison of nursing minimal data sets. J Am Med Inform Assoc. 1998;5(2):152-163. [doi: 10.1136/jamia.1998.0050152] [Medline: 9524348]

37. Wells JL, Egan M, Byrne K, Jaglal S, Dumbrell AC, Stolee P. Uses of the National Rehabilitation Reporting System: perspectives of geriatric rehabilitation clinicians. Can J Occup Ther. Oct 2009;76(4):294-298. [doi: 10.1177/000841740907600408] [Medline: 19891299]

40. Salva A, Corman B, Andrieu S, et al. Minimum data set for nutritional intervention studies in the elderly IAG/ IANA task force consensus. J Nutr Health Aging. 2004;8(4):202-206. [Medline: 15316582]

45. Dougherty M, Mitchell S. Getting better data from the MDS. Improving diagnostic data reporting in long-term care facilities. J AHIMA. 2004;75(10):28-33. [Medline: 15559836]

46. Martin CM. Getting ready for MDS 3.0: patient evaluation takes a new turn. Consult Pharm. Jul 2010;25(7):404-406. [doi: 10.4140/TCP.n.2010.404] [Medline: 20601346]

48. Buhr G, White HK. MDS 3.0 perspective: a better tool for patient care. J Am Med Dir Assoc. Mar 2013;14(3):221-222. [doi: 10.1016/j.jamda.2012.11.014] [Medline: 23318045]

50. Morley JE. Minimum data set 3.0: a giant step forward. J Am Med Dir Assoc. Jan 2013;14(1):1-3. [doi: 10.1016/j.jamda.2012.10.014] [Medline: 23200806]

52. Rahman AN, Applebaum RA. The nursing home minimum data set assessment instrument: manifest functions and unintended consequences--past, present, and future. Gerontologist. Dec 2009;49(6):727-735. [doi: 10.1093/geront/gnp066] [Medline: 19531805]
